# Supplementary figures and images for: The High Osmolarity Glycerol Mitogen-Activated Protein Kinase regulates glucose catabolite repression in filamentous fungi
Source: PLoS Genet. 2020 Aug 25;16(8):e1008996. doi: 10.1371/journal.pgen.1008996 (PMC7473523; doi:10.1371/journal.pgen.1008996)

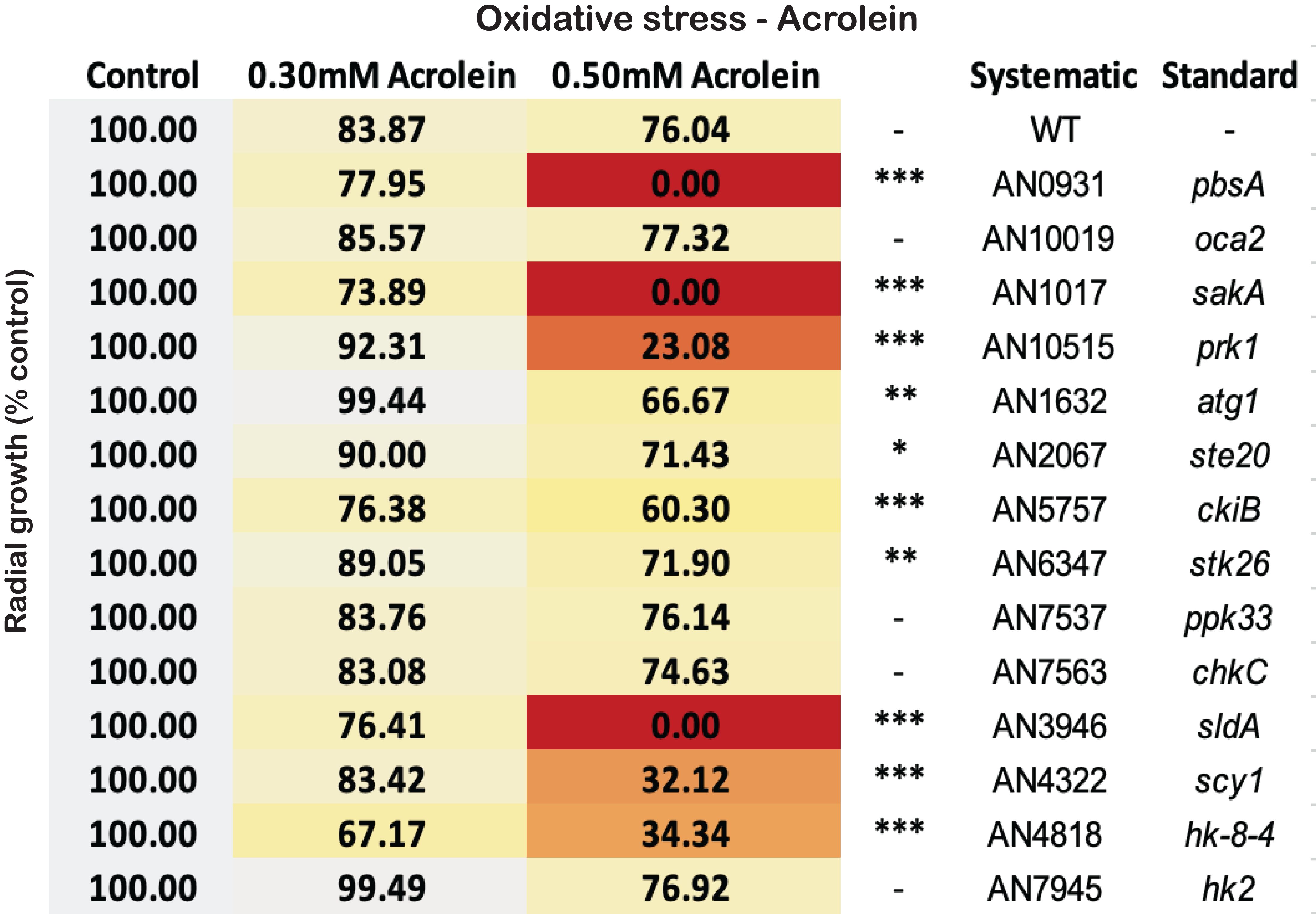

Supplement: S1 Fig — Heat map and values depicting average radial growth of three biological replicates of protein kinase deletion strains that were significantly sensitive or resistant to at least one concentration of acrolein. Strains were grown from 105 spores for 5 days at 37°C before radial diameter was measured. The results are expressed as percentage of growth in the presence of acrolein when compared to the drug-free, control medium (defined as 100% growth for each strain). Statistical analysis was performed using a one-tailed, paired t-test when compared to the control condition (*, p < 0.01 and **, p < 0.001). (TIF) [file pgen.1008996.s001.tif]

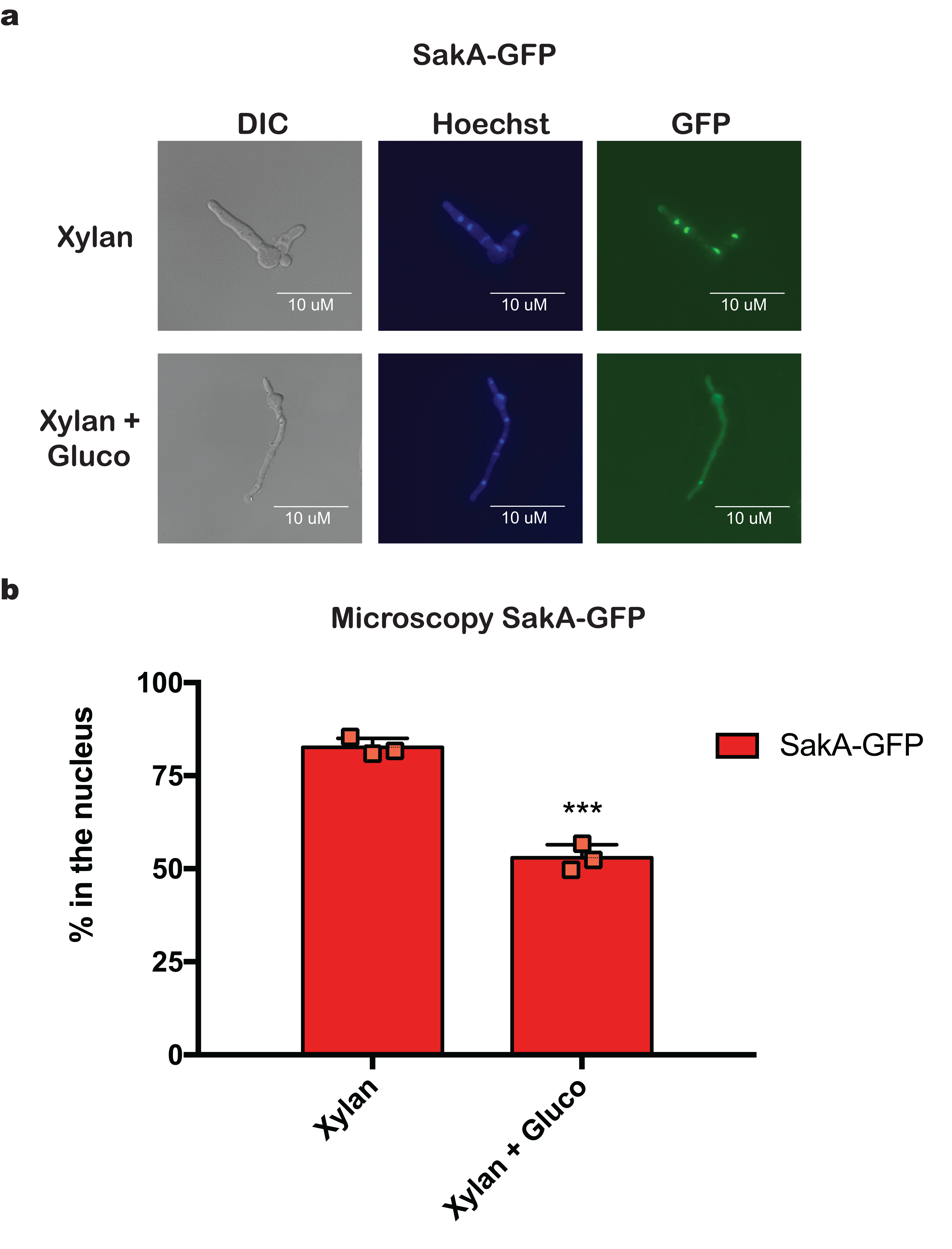

Supplement: S2 Fig — Microscopy pictures of SakA-GFP hyphae, taken after 16 h growth at 22°C in xylan minimal medium (MM) and after the addition of glucose for 30 min, show localization in the nucleus. Pictures were taken at different wavelengths (DIC = differential interference contrast, GFP = green fluorescent protein, Hoechst = Hoechst 33258 nucleic acid stain and merged) and scale bars are indicated (a). Percentage of SakA-GFP nuclear localization in different conditions. SakA-GFP was grown as specified in (a) before nuclei with and without GFP were counted for 100 hyphal germlings for each condition and the % of SakA-GFP localization was calculated. Hyphae were stained with Hoechst 33258 in order to confirm GFP nuclear localization (b). (TIF) [file pgen.1008996.s002.tif]

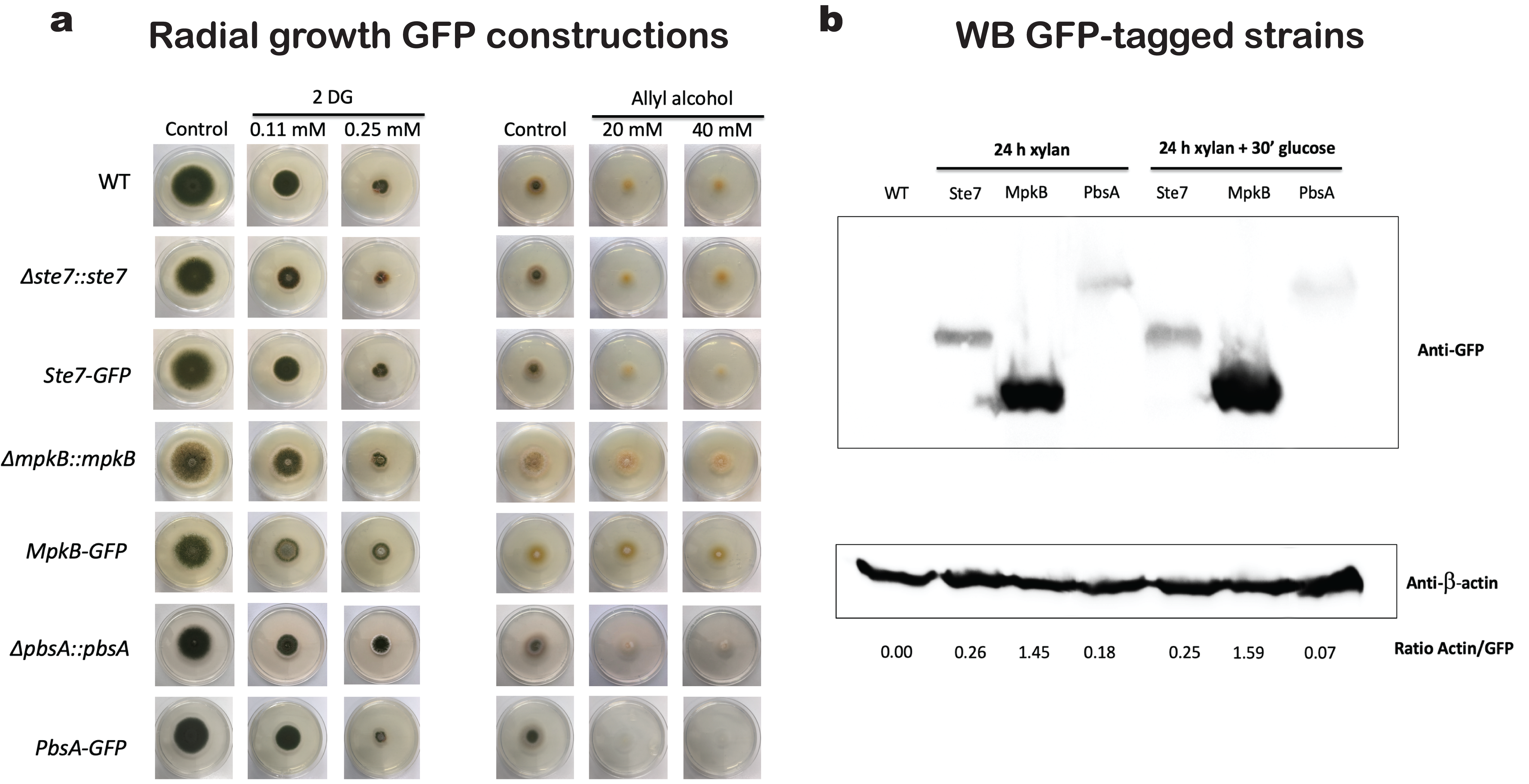

Supplement: S3 Fig — The Δste7, ΔmpkB and ΔpbsA strains were complemented with ste7-gfp, mpkB-gfp and pbsA-gfp respectively, and the same GFP-constructions were transformed into the wild-type (WT) background strain. Strains were grown from 105 spores on xylose or glucose minimal medium (MM) supplemented with increasing concentrations of 2-deoxy-glucose (2DG) and allyl alcohol (AA), respectively, for 5 days at 37°C before pictures were taken (a). The presence of full length GFP-tagged proteins was confirmed by Western blot after 24 h growth in xylan (carbon catabolite de-repressing condition) MM and after the addition of glucose (carbon catabolite repressing condition) for 30 min. Protein levels were normalized by β-actin (GFP/β-actin ratios are indicated) (b). (TIF) [file pgen.1008996.s003.tif]

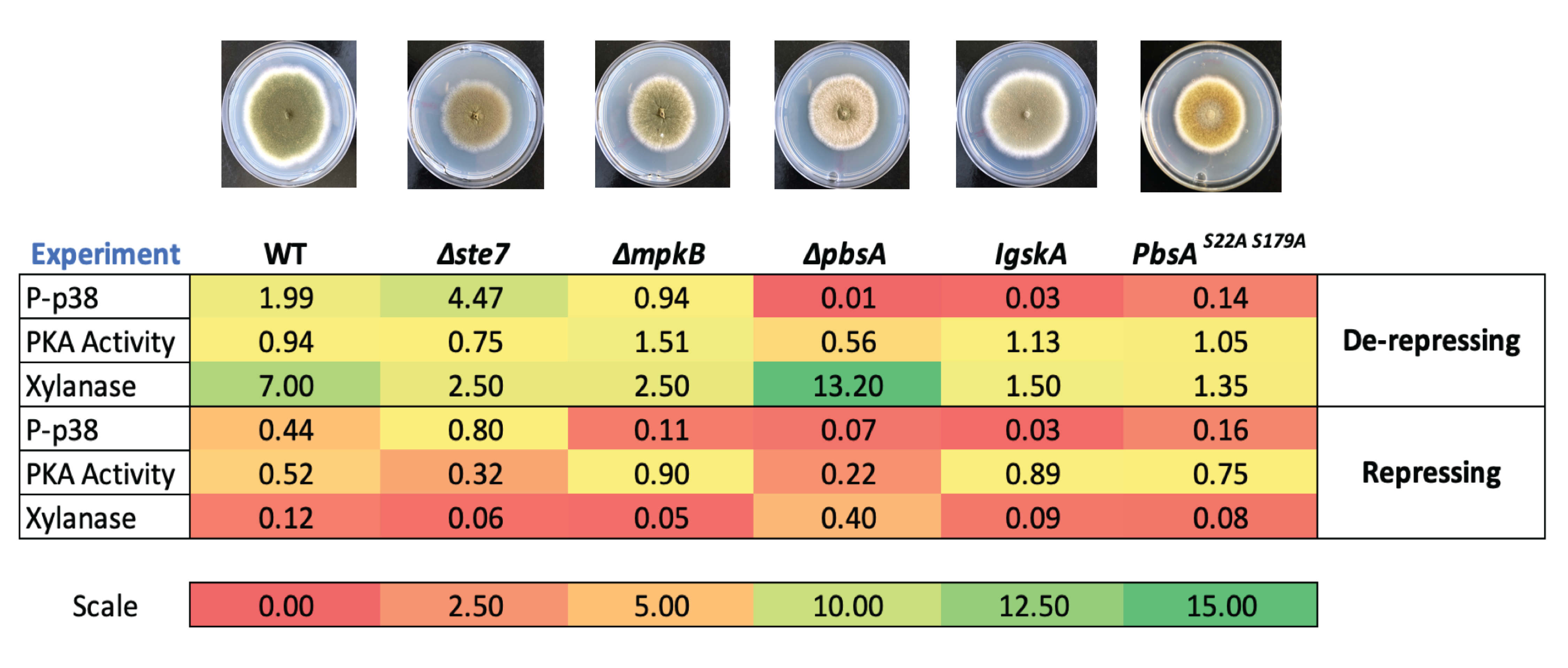

Supplement: S4 Fig — Heat map depicting SakA phosphorylation levels (P-p38/actin ratio), PKA activity (proportional arbitrary units), xylanase activity (mU x mL x mg dry weight) in the WT, Δste7, ΔmpkB, ΔpbsA, IgskA and PbsAS22A S179A strains in the presence of carbon catabolite (CC)-de-repressing and CC-repressing conditions. Numbers represent the average of the results from at least three biological replicates and the heat map color scale is also indicated. Also shown are representative images of the radial growth for each strain in the presence of glucose minimal medium. (TIF) [file pgen.1008996.s004.tif]
