# Supplementary material for: The High Osmolarity Glycerol Mitogen-Activated Protein Kinase regulates glucose catabolite repression in filamentous fungi
Source: PLoS Genet. 2020 Aug 25;16(8):e1008996. doi: 10.1371/journal.pgen.1008996 (PMC7473523; doi:10.1371/journal.pgen.1008996)
Supplement: S2 Table — (DOCX) [file pgen.1008996.s006.docx]

| **Strain name** | **Genotype** |
| --- | --- |
| AGB551 | Wild-type *﻿nkuAΔ::argB; pyroA4^-^; pyrg89^-^; veA^+^* |
| *∆ste7* | *ste7* (*mkkB*)::ptrA; *﻿nkuAΔ::argB; pyroA4^-^; pyrg89^-^; veA^+^* |
| C*ste7 (complem)* | *∆ste7* (*mkkB*)::ptrA Ste7(mkkB-GFP) Nat^+^;*﻿ nkuAΔ::argB; pyroA4^-^; pyrg89^-^; veA^+^* |
| Ste7-GFP | *ste7* (*mkkB*)-GFP Nat^+^; *﻿nkuAΔ::argB; pyroA4^-^; pyrg89^-^; veA^+^* |
| *∆mpkB* | *mpkB* (*Fus3*)::ptrA paba^-^; *nkuAΔ::argB;* yA2; *veA^+^* |
| C*mpkB* *(complem)* | *∆mpkB* (*Fus3*)::ptrA mpkB(Fus3)-GFP Nat^+^; *nkuAΔ::argB;* paba^-^; yA2 *veA^+^* |
| MpkB-GFP | *mpkB* (*Fus3*)-GFP VeA^+^ Nat^+^; *nkuAΔ::argB; pyroA4^-^; pyrg89^-^; veA^+^* |
| *∆pbsA* | *pbsA*::Phleo *nkuAΔ::argB; pyroA4^-^; pyrg89^-^; veA^+^* |
| C*pbsA* *(complem)* | *∆pbsA*::Phleo PbsA-GFP (pyro marker) *nkuAΔ::argB; pyrg89^-^; veA^+^* |
| PbsA-GFP | PbsA-GFP (pyroA marker) *nkuAΔ::argB; pyrg89^-^; veA^+^* |
| PbsA^S22A S179A^-GFP | PbsA-GFP S22A S179A (pyroA marker) *nkuAΔ::argB; pyrg89^-^; veA^+^* |
| CreA-GFP | *creA*::CreA-GFP *pyroA4* *nkuAΔ::argB; pyrg89^-^; veA^+^* |
| *∆ste7* CreA-GFP | *creA*::CreA-GFP *pyroA4* ste7 (*mkkB*)::ptrA; *﻿nkuAΔ::argB; pyrg89^-^; veA^+^* |
| *∆mpkB* CreA-GFP | *creA*::CreA-GFP *pyroA4* *mpkB* (*Fus3*)::ptrA; *nkuAΔ::argB; pyrg89^-^; veA^+^* |
| *∆pbsA* CreA-GFP | *creA*::CreA-GFP *pyroA4* *pbsA*::Phleo *nkuAΔ::argB; pyrg89^-^; veA^+^* |
| GFP OE | *pGPDH-GFP Nat^+^; pabaA1; nkuAΔ::argB; yA2 veA^+^* |
| SakA-GFP | *﻿nkuAΔ::argB pabaA1 sakA-GFP::pyroA^+^ pyroA4 veA^+^* |
