# Supplementary material for: The High Osmolarity Glycerol Mitogen-Activated Protein Kinase regulates glucose catabolite repression in filamentous fungi
Source: PLoS Genet. 2020 Aug 25;16(8):e1008996. doi: 10.1371/journal.pgen.1008996 (PMC7473523; doi:10.1371/journal.pgen.1008996)
Supplement: Attachment — Submitted filename: Answers to the Reviewers.docx [file pgen.1008996.s007.docx]

Answers to the Reviewers

Reviewer #1:

1) Carbon catabolite repression CCR, is an important regulatory process in fungi and bacteria and is of particular important in fungal biology. Here the authors utilized a protein kinase deletion collection in the fungus Aspergillus nidulans to identify protein kinases involved in CCR. The work complements existing observations linking protein kinase activity and regulation of CCR in many organisms. The manuscript contains a significant amount of data that concludes the HOG pathway regulates CCR in fungi. While the conclusions are reasonable based on the data presented, some experiments are missing key controls and leave alternative conclusions in play. Some methods used are notorious for giving spurious results (e.g. kinase assay kits). Also - difference between 2-DG and AA results seem to not be considered as both impact stress responses in the cell beyond CCR that likely are mediated by HOG and related kinases. A lot of emphasis is placed on single assays such as CreA-GFP localization and phosphorylation of SakA. Additional evidence of SakA activation and CreA activity are needed to improve the rigor of the studies.

Answer: We thank the reviewer for the comments and suggestions. To address the impact of 2DG and AA on other cellular stress responses we have added the following sentences and experiments (Pages 7 and 8, lines 174 and 175 to 182, respectively) “In the cell, AA is converted to acrolein, a potent inducer of oxidative stress [39–41]. To determine whether the observed strain phenotypes could also be related to defects in the oxidative stress response, we assessed resistance and sensitivity of these strains to acrolein (Fig. S1). Strains Δ*pbsA*, Δ*sakA*, Δ*prk1*, Δ*atg1*, Δ*ste20*, Δ*ckiB*, Δ*stk26*, Δ*sldA*, Δ*scyA*, and Δ*hk-8-4* were sensitive to increased concentrations of acrolein (Fig. S1). These strains, with the exception of the ΔckiB and Δhk-8-4 strains were also sensitive to AA (Fig. S1). These results therefore suggest that the observed strain resistance/sensitivity phenotypes to AA could be due to either defects in CCR and/or oxidative stress responses”.

And

(Page Lines 187-190): “It has been shown that increased resistance or sensitivity to 2DG can be due to a reduction in glucose uptake in a mutation-dependent manner [36,37] in addition to other 2-DG off-target effects [38].”

To provide additional evidence for SakA activation and CreA activity we have carried out the following experiments: i) We have performed a Western blot for SakA phosphorylation on the Δ*sakA* strain and show that no signal was obtained (negative control, Fig. 2b), showing the specificity of this antibody. Unfortunately, we were unable to use an antibody against total cellular SakA as this antibody does not work in *A. nidulans* and used an antibody against beta-actin instead. ii) We have measured PKA activity in the Δ*pkaA* strain (deleted for the main PKA catalytic subunit, Fig. 2c) and show that activity was significantly lower in this strain than in the other strains, suggesting that our PKA assay is specific enough to differentiate between strains with impaired PKA activity versus strains with no PKA activity. iii) In Fig. 4a, Western blot normalization was carried out by beta-actin levels (CreA-GFP full length/beta-actin ratios were also calculated and are indicated) and the Coomassie blue normalization gel was removed. iv) We also performed SakA-GFP microscopy in CC-de-repressing and repressing conditions and show that this MAPK is predominantly nuclear in the presence of xylan and localizes to the cytoplasm in the presence of glucose (Fig. S2, page 10, lines 238-245). This is further evidence for this MAPK playing a role in carbon source utilization in *A. nidulans*.

2) Figure 1 data is important for the study – but some consideration discussion of 2-DG off-target effects and AA generation of oxidative stress should be considered. Are AA R/S strains also R/S to oxidative stress?

Answer: We thank the reviewer for the suggestion. To address the impact of 2DG and AA on other cellular stress responses we have grown the strains in the presence of the oxidative stress-inducing compound acrolein (and product of AA metabolism) and added the following sentences(Pages 7 and 8, lines 174 and 175 to 182, respectively) “In the cell, AA is converted to acrolein, a potent inducer of oxidative stress [39–41]. To determine whether the observed strain phenotypes could also be related to defects in the oxidative stress response, we assessed resistance and sensitivity of these strains to acrolein (Fig. S1). Strains Δ*pbsA*, Δ*sakA*, Δ*prk1*, Δ*atg1*, Δ*ste20*, Δ*ckiB*, Δ*stk26*, Δ*sldA*, Δ*scyA*, and Δ*hk-8-4* were sensitive to increased concentrations of acrolein (Fig. S1). These strains, with the exception of the Δ*ckiB* and Δ*hk-8-4* strains were also sensitive to AA (Fig. S1). These results therefore suggest that the observed strain resistance/sensitivity phenotypes to AA could be due to either defects in CCR and/or oxidative stress responses”.

And

(Page Lines 187-190): “It has been shown that increased resistance or sensitivity to 2DG can be due to a reduction in glucose uptake in a mutation-dependent manner [36,37] in addition to other 2-DG off-target effects [38].”

3) The assay to measure glucose uptake is not a true glucose uptake assay. The author’s measured glucose in the culture media after incubation with the fungus. The changes in glucose levels in the media may be due to differences in uptake, but also maybe not. To measure uptake, radio-labeled glucose is needed. Since the mutants have growth defects, it is unclear if the differences in the media are simply due to the reduced growth of the mutants and lack of glucose utilization.

Answer: We thank the reviewer for the observation. The reviewer is absolutely right. The word “uptake” was replaced by “transport”, and in the Methods section, the methodology is now described as “Detection of glucose in the supernatant” (Pages 25 and 26, lines 619 to 622 and 623 to 626, respectively). The data is presented here as a percentage of glucose in comparison to the control condition for each strain. Strains were grown in complete medium prior to transfer to glucose minimal medium and measuring glucose concentrations in the supernatants, in order to reduce strain-specific growth defects. Finally, the head of the Figure 1d is now described as “Glucose transport” (instead of previously “Glucose uptake”)

To address the possibility of growth defects affecting glucose transport we have added the following sentence (Page 8, lines 195 to 198): “These results suggest that the significant reduction in glucose transport in the *ΔpbsA* and *ΔsakA* may be related to growth impairments in this carbon source. Nevertheless, several MAPK pathway kinases appear to be important for glucose metabolism in *A. nidulans*.”

4) Fig. 2A – unclear what the STRING analyses is presenting. Are these direct physical interactions? Indirect genetic interactions? More details on this analysis are needed.

Answer: We thank the reviewer for the observation. The STRING analysis shows both direct physical and/or indirect genetic interactions and is a summary of published interactions that are taking place between proteins. We have added to Fig. 2A a small legend explaining the meaning of the arrows (direct/indirect physical and/or genetic interaction) and have also added this information in the legend of the figure.

5) For the western blots of SakA phosphorylation – total SakA is a critical control that is missing. Without that control, interpretation of these data are tenuous. Also, a sakA null mutant is an important control here.

Answer: We have performed a Western blot for SakA phosphorylation in the *ΔsakA* strain and show that no signal was obtained (negative control, Fig. 2b), supporting the specificity of this antibody (lines 218-220). We also provide the ratios of phosphorylated SakA levels and beta-actin levels as a signal normalization value. Unfortunately, we were unable to use an antibody against total cellular SakA as this antibody does not work in *A. nidulans* and used an antibody against beta-actin instead. We have tried several antibody brands for the antibody that recognizes total SakA protein and none of them has worked in our hands.

6) Lines 204/205 – Xylose is listed as being both de-repressing and repressing

Answer: The sentence was changed to (Page 9, lines 223 to 224): “...when grown in CC-de-repressing (xylose) and CCR (in the presence of glucose) conditions.”

7) Is phosphorylation of SakA sufficient evidence to conclude that activation is happening or inhibited in these conditions? Does SakA localization change in repressing and de-repressing conditions?

Answer: SakA phosphorylation has generally been perceived as the signal for HOG pathway activation in a number of fungi and conditions. We thank the reviewer for the suggestion on SakA cellular localization. We performed SakA-GFP microscopy in CC-de-repressing and repressing conditions and show that this MAPK is predominantly nuclear in the presence of xylan and localizes to the cytoplasm in the presence of glucose (Fig. S2, page 10, lines 238 to 247-240): “In addition, we determined cellular localization of SakA in CC-de-repressing (xylan) and repressing (xylan and glucose) conditions by constructing a strain with a single copy of SakA-GFP integrated at the *sakA* locus and under the control of the native promoter. In the presence of xylan, SakA-GFP was predominantly observed in the nuclei (82.6 %); whereas the addition of 2% w/v glucose for 30 min, resulted in the partial re-localization of SakA-GFP to the cytoplasm with SakA-GFP fluorescence observed in 52.9 % of nuclei (Fig. S2). These results suggest that SakA cellular localization is carbon source-dependent and important for the control of carbon catabolite de-repression in *A. nidulans*.”

8) Kinase activity assays such as those in Figure 2C need mutant controls – these assays are notorious for non-specific kinase activity. What is the PKA activity in a PKA mutant strain?

Answer: We have measured PKA activity in the Δ*pkaA* strain (deleted for the main PKA catalytic subunit, Fig. 2c) and show that activity was significantly lower in this strain than in the other strains, suggesting that our PKA assay is specific enough to differentiate between strains with impaired PKA activity versus strains with no PKA activity.

9) Figure 3A – these are not the best sub-cellular localization images. Are they representative? The nuclei are not clear and no co-localization is shown.

Answer: We added other sub-cellular localization images that are representative for the microscopy that was performed. In support of the images is Figure 3b which contains the averages and standard deviations of 3 biological replicates and assessment of around 100 germlings. Unfortunately, we do not have an appropriate filter in our fluorescent microscope to show a high-quality merged image, so we’ve removed the merged image from the figure.

10) Figure 4A – approximate size of bands needed; loading control is hard to read, why not use the actin antibody here? There seems to be a lot of variability in protein loaded.

Answer: We have add the approximate size of bands to the figure and replaced Coomassie stained gel of the input samples by a Western blot with anti-B-actin.

11) Figure 5 – these are important data, but they are preliminary without confirmation via rigorous Co-IP experiments and for the phosphoproteomics, confirmation that the phosphosites are indeed functional.

Answer: We do agree with the reviewer that these results are actually only preliminary results that need further validation. In our manuscript, we have started the validation by mutating the phosphorylated residues in PbsA. However, further validation of other phosphorylation sites and interacting partners is beyond the scope of this manuscript.

12) has this gskA inhibitor been validated in fungi? Perhaps an inducible gskA strain is a more rigorous approach to support the conclusions.

Answer: This GskA inhibitor has already been validated in fungi. We have added a sentence to the manuscript stating that (Pages 16 and 17,line 398 to 399): “Similar Gsk3 inhibitor compounds have already been shown as inhibiting *A. nidulans* growth [50].”

13) How do changes in carbon sources used in the study impact the osmolarity of the system? Is this activation of HOG really reflecting changes in osmolarity?

Answer: The changes in carbon sources concentrations are not dramatic. However, as can be seen in Figure 2b, when we added 2% glucose to the 1% xylose-grown cultures (resulting in an increase in the osmolarity), there is a decrease in SakA phosphorylation. Thus, it is unlikely that the activation of HOG is really reflecting changes in osmolarity.

Reviewer #2

The authors started off with a mutant collection of non-essential protein kinases in Aspergillus nidulans because the activity of the main regulator of carbon catabolite repression, CreA, is regulated by phosphorylation. Studying potential roles in carbon catabolite repression they discovered that the central regulator CreA is able to form protein complexes with several other regulators. Apparently, the protein complex is a scavenger for free CreA, which upon dissociation can fulfill its function. This work nicely demonstrates the cross-talk between carbon utilization and different signaling pathways in the cell.

This is one of the few papers where I don’t have any major suggestions for improvement. Excellent paper. However, I am not sure whether the proposed large protein complex as displayed in Fig. 8 is not an oversimplification. I don’t think the data really show that such a large protein complex exists. The observed interactions could be transient interactions of different protein combinations. Or did I oversee something?

Answer: We thank the reviewer for the comments and suggestions and absolutely agree with them. Accordingly, we have modified relevant paragraphs in the manuscript to further emphasize the possibility of transient interactions (Pages 15 and 16, lines 364 to 373 and 374 to 388, respectively). In addition, Fig. 8 was modified and all transient complexes identified during the Ste7-GFP, MpkB-GFP and PbsA-GFP pull downs were added to the diagram. For clarity, regulatory subunits were removed from PkaA and CreA and SakA was also added in the nucleus during de-repressing conditions (confirmed after performing SakA-GFP microscopy; Fig. S2).

Reviewer #3:

In fungal organisms, carbon catabolite repression (CCR) is a regulatory mechanism that inhibits enzymes for the consumption of secondary carbon sources, when a preferred primary carbon source such as glucose is present. CCR has been studied for long in diverse fungal genetic model systems, including Aspergillus nidulans. This for example led to the discovery of transcription factor (TF) CreA, which is highly conserved in fungal organisms. The regulation of CreA is mediated by phosphorylation at different residues. This complexity of the regulation of carbon source utilisations is only partly understood, including the interplay between different kinase signaling pathways.
In this paper, different mitogen-activated protein kinases (MAPKs) were identified that are crucial for CCR and protein kinase A (PKA) activity, which is essential for carbon source utilisation. With this, the manuscript contributes substantially to our understanding of the crosstalk between different signaling pathways.
The paper starts with the screening of a library, containing non-essential protein kinase (NPK) deletion strains, to identify kinases important for CCR. This resulted in the identification of several MAPK pathway kinases. This is followed by construction of a network interaction profile of 23 protein kinases, and a microscopic investigation of MAPKs dependent nuclear CreA localization. Finally, MS and phosphoproteome analysis identifies interaction partner of MAPKS and the effect of MAPK phosphorylation on osmotic stress resistance, HOG pathway activation and enzyme activities.
At the end of the ms, the authors contribute a model, explaining the correct integration of signaling events and cascades that constitute the different pathways involved in glucose-mediated CCR as well as CC-de-repression.
The data presented are of high quality and the ms addresses the major question how different carbon sources are utilized on the molecular level. As such, the ms deserves publication. However, I would like to suggest several improvements

Answer: We thank the reviewer for the comments and suggestions.

1. Important information is missing in the Methods section: the construction of phosphorylation mutants (line 388) should be included. In case of Data Processing (line 633 - 638), I recommend to give the following information in the Methods section:

Answer: We have added more detailed information on the methodology for the construction of the phosphomutants (Pages 24 and 25, lines 588 to 597 and 598 to 603, respectively): “The strain harboring a double point mutation in *pbsA*, where serines 22 and 179 were mutated to an alanine, was constructed by replacing the endogenous gene by the respective *pbsA*^S22A S179A^-*gfp* cassette. gDNA from the PbsA-GFP (5’UTR-*pbsA*-*gfp*-*AfpyrG*-3’UTR) strain was used as a template for subsequent PCR amplifications. The following three fragments were generated: a) 5’UTR-*pbsA* (partial sequence until the first point mutation), b) *pbsA* fragment carrying the two mutations S22A S179A (using primers containing the mutations and 20 bp flanking sequence) and c) *pbsA* (partial sequence after the second point mutation)-*gfp*-*AfpyrG*-3’UTR. The three fragments were recombined into one cassette using plasmid pRS426 and transformed into *Saccharomyces cerevisiae* strain SC9721 (MATα his3-Δ200 URA3-52 leu2Δ1 lys2Δ202 trp1Δ63) obtained from the Fungal Genetic Stock Center (FGSC). All plasmids were cloned into bacteria before full cassettes were amplified by PCR from extracted bacterial plasmid DNA (Qiagen Plasmid miniprep) and used for transformation in *Aspergillus nidulans* as previously described. The presence of the *pbsA* double point mutation was confirmed by DNA sequencing.”

- Database size and origin (e.g. Uniprot, Refseq?)

Answer: AspGD (Aspergillus Genome Database) to obtain functional annotations, which consist of 29873 entries for the *A. nidulans* FGSC A4 genome (Page 29, lines 702 to 706): “We have used the AspGD (Aspergillus Genome Database) to obtain functional annotations, which consist of 29873 entries for the *A. nidulans* FGSC A4 genome. STY phosphorylation was considered as a variable modification, and phospoRS was used to calculate site probabilities with a cut off value of 0.8 [60].”

- Precursor and fragment mass tolerance

Answer: Precursor mass tolerance, 10 ppm; Fragment mass tolerance, 0.6 Da (Page 28, lines 695to 696): “The precursor mass tolerance was 10 ppm while the fragment tolerance was 0.6 Da.”

- Enzyme specificity

Answer: Trypsin (Full), Maximum missed cleavage sites, 2 (Pages 28 and 29, lines 696 and 697 to 698, respectively): “The experiments had full trypsin enzyme specificity with 2 as a maximum missed cleavage sites, the FDR target value was 0.01 PSMs.”

- Which FDR target value was used and to which level was it applied (PSMs, Peptides, Proteins)?

Answer: 0.01 for all (Pages 28 and 29, lines 696 and 697 to 698, respectively): “The experiments had full trypsin enzyme specificity with 2 as a maximum missed cleavage sites, the FDR target value was 0.01 PSMs.”

- Was a phosphoRS cutoff value applied?

Answer: ptmRS scores are part of the tables we sent. We set 0.8 cutoff (Page 29, lines 704 to 706): “STY phosphorylation was considered as a variable modification, and phospoRS was used to calculate site probabilities with a cut off value of 0.8 [60].

2. line 249ff: The experiments for Fig. 4a should be described in more detail. For example, carry all strains single copies of the gfp-creA construct? Further, how were the CreA-GFP protein levels measured? Did the authors take also in account the CreA-GFP degradation products, or did they consider only the full size CreA-GFP protein? One may conclude from the protein gel that single lanes contain different protein amounts.

Answer: We thank the reviewer for the suggestions. The sentences were modified to (Page 11, lines 267 to 268): ”All these strains have single copies of CreA:GFP integrated at the *creA* locus and under the control of the native promoter.”,

and

We took into consideration only the main band of CreA-GFP indicated by an arrow in Figure 4A (Page 12, lines 282 to 286): “Similarly to a previous study [31], CreA-GFP protein levels (about 76 KDa, indicated by an arrow in Fig. 4a) are reduced in the presence of xylose (CC-de-repression) in the WT strain, whereas the addition of glucose (CCR) caused an increase in CreA-GFP protein levels (Fig. 4a).”

We have replaced Coomassie staining of the input by a Western blot for anti-B-actin.

3. I miss mentioning PkaC and PkaR, both which are present in the model of Figure 8, but are not described in the ms.

Answer: The reviewer is right and PkaR was removed from the Figure 8. We have added it before because *A. fumigatus* PkaR-PkaA complex formation during CC de-repressing and –repressing conditions has previously been published (Ribeiro, L.F.C *et al* 2019 mBio).

4. I suggest to give a list of abbreviations of the many genes/proteins for better readability of the MS

Answer: We thank the reviewer for the comment. Table S1 contains the systematic name, standard name and description of each protein (and corresponding gene ID) identified in the MS data.

5. Some formal points:

- Fig. 3a: It should read “Hoechst”, not Hoescht

Answer: This has been corrected.

- Fig. 3b: Why do they show a 125% y-axis? More than 100 % should be impossible. It should read “Microscopy”-

Answer: The y-axis was changed to 100 % and the Word “Microscopy” was corrected.

Fig. 5d: “Prediction”, instead of “Predition”

Answer: This has now been corrected.

- Check the list of references, which is not consistently abbreviated, e.g. Fungal Gen Biol or Fungal Genet Biol or Fungal Genet Biol. Elsevier Inc.

Answer: The reference list has been checked and consistent abbreviations are now given.
